# Group Prenatal Care in Mexico: perspectives and experiences of health personnel

**DOI:** 10.11606/s1518-8787.2020054002175

**Published:** 2020-11-27

**Authors:** Midiam Ibañez-Cuevas, Ileana Beatriz Heredia-Pi, Evelyn Fuentes-Rivera, Zafiro Andrade-Romo, Jacqueline Alcalde-Rabanal, Lourdes Bravo-Bolaños Cacho, Xochitl Guzmán-Delgado, Laurie Jurkiewicz, Blair G Darney

**Affiliations:** I Instituto Nacional de Salud Pública Centro de Investigación en Sistemas de Salud CuernavacaMOR México Instituto Nacional de Salud Pública. Centro de Investigación en Sistemas de Salud. Cuernavaca, MOR, México; II University of California San Francisco San Francisco General Hospital Department of ObGyn & Reproductive Sciences San FranciscoCA USA University of California San Francisco. San Francisco General Hospital. Department of ObGyn & Reproductive Sciences. San Francisco, CA, USA; III Oregon Health & Science University Department of Obstetrics & Gynecology PortlandOR USA Oregon Health & Science University. Department of Obstetrics & Gynecology. Portland, OR, USA

**Keywords:** Prenatal Care, Maternal-Child Health Services, organization & administration, Primary Health Care, Qualitative Research

## Abstract

**OBJECTIVE:**

Identify barriers and facilitators to implementing the Group Prenatal Care model in Mexico (GPC) from the health care personnel's perspective.

**METHODS:**

We carried out a qualitative descriptive study in four clinics of the Ministry of Health in two states of Mexico (Morelos and Hidalgo) from June 2016 to August 2018. We conducted 11 semi-structured interviews with health care service providers, and we examined their perceptions and experiences during the implementation of the GPC model. We identified the barriers and facilitators for its adoption in two dimensions: a) structural (space, resources, health personnel, patient volume, community) and b) attitudinal (motivation, leadership, acceptability, address problems, work atmosphere and communication).

**RESULTS:**

The most relevant barriers reported at the structural level were the availability of physical space in health units and the work overload of health personnel. We identified the difficulty in adopting a less hierarchical relationship during the pregnant women's care at the attitudinal level. The main facilitator at the attitudinal level was the acceptability that providers had of the model. One specific finding for Mexico's implementation context was the resistance to change the doctor-patient relationship; it is difficult to abandon the prevailing hierarchical model and change to a more horizontal relationship with pregnant women.

**CONCLUSION:**

Analyzing the GPC model's implementation in Mexico, from the health care personnel's perspective, has revealed barriers and facilitators similar to the experiences in other contexts. Future efforts to adopt the model should focus on timely attention to identified barriers, especially those identified in the attitudinal dimension that can be modified by regular health care personnel training.

## INTRODUCTION

Significant challenges remain in maternal health regarding access and quality of care in Mexico and in many Latin American countries. The mainly affected populations have a greater structural and social vulnerability, lower health service coverage and less probability of receiving adequate prenatal care ^1^^-^^3^ . The standard model of individual prenatal care has been widely questioned because of long waiting times, gaps in continuity of care, and low satisfaction of patients with staff treatment ^4^^,^^5^ .

The World Health Organization has issued recommendations on prenatal care for a positive experience during pregnancy. The recommendations refer to health system interventions that improve the utilization and quality of prenatal care ^6^ . The implementation of an innovative model has been suggested to improve maternal and child outcomes in specific contexts. This model is the Group Prenatal Care (GPC) model, which offers an alternative to individual prenatal care. The model is characterized by integrating components of self-care, clinical review, health education and construction of social support networks ^7^ .

Prenatal care is provided by various health care providers who assume a facilitating role in this model. Groups of between eight and twelve women of similar gestational age (12–20 weeks) are organized into small cohorts that meet eight to ten times during pregnancy in sessions of about two hours, incorporating all the components mentioned in a circuit of four phases ^8^ . Several GPC models, including CenteringPregnancy, the most widespread and studied group model, have been implemented worldwide in industrialized and low and middle-income countries and specific populations ^4^^,^^9^^-^^11^ .

**Figura 1:**
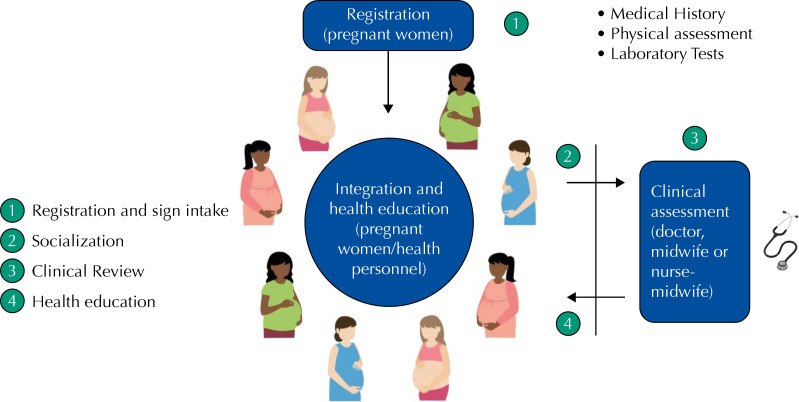
Ciclo de atención en el modelo de Atención Prenatal en Grupo. México, 2016–2018

The different phases of the cycle of care in each of the group sessions of group prenatal care, based on the CenteringPregnancy model include: (1) registration and self-care: participants start the session and check their vital signs; (2) socializing or building social networks: they sit in a circle and spend time talking freely with their peers; (3) medical check-up: in parallel with the second phase, each woman has an individual consultation with the doctor or midwife, who performs individual physical exams, in the same place where the group meets; and (4) health education: once the round of physical examinations is completed, women and facilitators come together into the same circle, where information about pregnancy is shared between them in a participatory, non-hierarchical approach ( Figure 1 ).

Fuente: Elaboración propia tomando como referencia al modelo CenteringPregnancy ^7^^,^^8^ .

The process of implementing innovative interventions such as GPC faces significant barriers that impact the stages of adoption and escalation, as well as the results that are achieved ^12^ . The GPC represents a change in the user-provider relationship and the dynamics and organization of the health units. Thus, it is relevant to explore the perceptions of health personnel and their experience with implementing interventions that change the way health services are offered. This analysis is essential to identify barriers and facilitators to the final adoption of the model within the health system and possible areas of opportunity for strengthening its implementation and future escalation.

Studies have explored the barriers and facilitators of GPC implementation, based on the perceptions of health personnel who took on the facilitator's role. In general, facilitators have positive perceptions and consider that the model has advantages over the individual care model. Such as more time for medical staff to interact with the pregnant woman, better relationships and communication with women, and the attention is much more satisfying and effective in improving health promotion ^13^^-^^19^ .

Among the challenges for the successful implementation of the model are the health system's support and leadership ^18^ . Also, staff motivation and communication within the health center ^12^ and flexibility and commitment are crucial elements for this stage ^20^ . Additionally, from the health personnel's perspective, different barriers to implementation have been identified, including distrust regarding the clinical review process in about three minutes, which, according to their experience, is insufficient ^16^ . Other obstacles are the lack of adequate physical space to conduct the sessions ^12^^,^^21^ , challenges in scheduling requiring coordinating the calendars of women, lack of health personnel and the use of the physical space ^11^^,^^18^^,^^20^ .

In Mexico, since 2016, we began a study to adapt the CenteringPregnancy model to the Mexican context and measure the feasibility and acceptability for women and health personnel during its initial implementation ^22^^,^^23^ . Given that this is the first time the GPC model was implemented in Mexico, it is relevant to know the experiences of the personnel who implemented it. To identify how much acceptance the model had among health service providers, how much they were involved during its implementation and the level of integration of the new intervention into the health units' context.

The objective of this study was, from the perspective of health personnel, to identify barriers and facilitators to the implementation of GPC model in Mexico.

## METHODS

**Figura 2:**
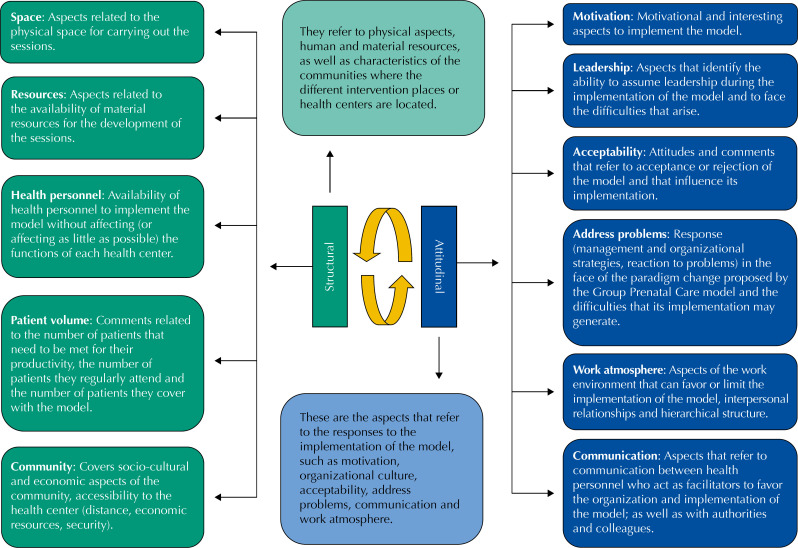
Marco conceptual y analítico. Dimensiones de exploración y categorías de análisis del modelo de Atención Prenatal en Grupo. México, 2016–2018.

We conducted a qualitative descriptive study in four health centers of the Ministry of Health in the states of Morelos and Hidalgo, Mexico. We used semi-structured interviews to explore health personnel's perceptions and identify barriers and facilitators of implementing the GPC model. The theoretical framework used as a guide for this research was the one suggested by Novick et al. ^12^ proposing two dimensions of exploration: a) structural (space, resources, health personnel, patient volume, community) and b) attitudinal (motivation, leadership, acceptability, address problems, work atmosphere and communication). In Figure 2 , the explored dimensions and categories of analysis are described. This conceptual proposal for analysis was based on exploring the perceptions of health personnel and used as a guide of the requirements or demands that the implementation of the model imposes on the institutions and health system ( Table 1 ). Factors favoring the system's capacity to meet these demands were identified as facilitators, while those hindering their achievement were considered barriers to their implementation ^12^ .

Fuente: Elaborado a partir de Novick et al. ^12^

**Table 1:** Essential elements and requirements for the development of the Group Prenatal Care model in Mexico, 2016-2018.

Essential element	Key points	Required elements
1.The medical examination takes place in the group space	Organize some elements in such a way that the area destined for the medical exam ensures privacy: place and level of the area, music, plants or some simple division	Enough space to maintain an area that allows the medical examinations to be done with privacy Supplies and equipment for physical exams (Doppler, measuring tape, prescription forms, pen, hand sanitizer, reference sheets, stethoscope) Resources for physical exams (mat or cot and screen -optional-) *
2. Women are involved in self-care activities	Women take and record some health parameters such as their blood pressure or weight	Specified space for women's self-evaluation Self-care instruments (scale, baumanometer, batteries, follow-up sheets, pens) * Pre-training of women (instructions to health personnel)
3. A facilitative leadership style is used	The facilitators guide, but do not control the discussion and refer questions to the group Women voluntarily share their experiences, feelings, ideas and information Rules are established for the group Facilitators dress informally Participants sign a confidentiality agreement	Educational materials (cards, boxes, scissors, yarn, flip chart, pens, etc.) * Formats (confidentiality agreement) * Enough space to have chairs for all assistants Chairs arranged in a circle Session guide (general plan or curriculum) * Training to health personnel focused on a horizontal facilitation style
4. Each session has an overall plan, although the emphasis may vary	Materials are used to guide and evaluate the session	Session guide (general plan or curriculum) Training for health personnel to make them aware of the flexibility of the model, emphasizing those elements that are essential
5. The group takes place in a circle	There are no observers outside the circle The space where the session takes place is private People sit in a circle in an open space Circle activities do not begin until all facilitators and women are in the circle	Enough space to have chairs for all assistants Chairs arranged in a circle
6. There is stability in the group members including the facilitators	New women can join the group as long as it is agreed by the members of the group Facilitators are present during all sessions There is a plan in case a facilitator cannot come If there are students supporting the group, they are supervised and consistent during all sessions. Women's children are not present during circle activities	Process and strategies for recruiting women at the facilitator and health center level Follow-up on women's attendance at sessions (reminders by phone or message) Adequate scheduling of sessions so that facilitators and women can be present for the entire session Definition of rules in a clear way with the group to determine aspects such as visitor attendance Training of two teams of facilitators per center to supply in case of any unforeseen event Training for health personnel with guaranteed work in the medium term
7. The size of the group is optimal to promote the process	The groups have 8-12 women There is an appropriate proportion of women and facilitators	Processes and strategies for recruiting women at the facilitator and health center level Follow-up on women's attendance at sessions (reminders by phone or message) Adequate scheduling of sessions so that facilitators and women can be present for the entire session
8. Opportunity for group socialization is provided	There is a free time when women can socialize with each other	Training that includes the relevance of networking processes among women Teams of facilitators of at least two people so that while one is doing the medical exams the other promotes the socialization of the women
9. There is a continuous evaluation of the results	There is a report of fidelity, health results and sustainability of the group Facilitators are constantly asking women how they feel about group prenatal care	Training should include elements of continuity and constant evaluation of progress and challenges

* Material provided by the research project.

We selected the participating health centers (HC) according to the patient volume, physical space availability to implement the model, and health personnel availability. Informants played the role of facilitators during the implementation of the model. The interviews lasted an average of 50 minutes and were conducted when the facilitator had completed the implementation of at least one GPC. Four of the 15 providers contacted did not agree to participate in the interview, arguing that they did not have enough time to conduct the interview.

We used a semi-structured interview guide that explored: a) sociodemographic data, b) perception about the model, c) barriers and facilitators for the development of the model, d) experience being a facilitator, and e) suggestions for improving the model. The guide was conducted with subjects close to the target population to achieve the instrument's internal validity.

We conducted 11 interviews with facilitators in the selected health centers. Most participants were women (8/11). Five interviews were with medical personnel, five with nurses and one with social work personnel. Five interviewees had 20-29 years of experience working in health services, four had 10-19 years of experience, and two had one to nine years.

We transcribed the interviews and then used a response matrix. We classified the information into the previously defined thematic components, corresponding to the proposed conceptual framework, to examine and describe their content, according to the conceptual framework's dimensions.

We carried out a thematic analysis identifying recurrences and coincidences in discourses, opinions and reported behaviors. Subsequently, we carried out the process of data interpretation. We checked the information's consistency and validity by identifying the testimonies offered iteratively by the participants. We carried out an exercise of interpretative triangulation ^24^ of the data between two researchers to guarantee its validity ^25^ .

Participants signed an informed consent form authorizing recordings of the interviews. The study was approved by the Ethics and Research Committees of the National Institute of Public Health of Mexico (No. 1756).

## RESULTS

The testimonies (T) that supported the findings identify the cited person by an interview number ( Table 2 ). We begin with a summary of the central findings for each category in the presentation of the results.

**Table 2:** Health personnel testimonials by dimension of the Prenatal Group Care model in Mexico, 2016-2018.

Category	Testimonials structural dimension
Space	**“** If we go to the structure because they are the spaces (...) in fact we don't have a place, they don't lend it to us, so I think that is going to be a very important difficulty (...) a space to be able to work both things and I don't know what the other colleagues think” **(E01, Physician, Clinic 1)** . **“** It is very difficult because we don't have the infrastructure to simply do the workshop (...) we don't have space and nothing but this health center, many health centers. The main thing is the infrastructure” **(E02, Nurse, Clinic 1)** .
Resources	**“** About the Doppler, I always said (...) because we only have one, so they would ask it. It didn't belong to the control prenatal group, it belongs to here [the health center], so when I got it, (...)they asked it me back” **(E04, Physician, Clinic 2)** . **“** Here we have (...) all the material, the tools. I don't know if it was you or who sent us the tool, which is a box with all the things [for the sessions].” **(E11, Nurse, Clinic 4)** .
Health Personnel	**“** What we did was the most feasible, occupying the time in the morning, when the cards are given, so, normally, we can give them or not. We used to distribute twenty-one cards. We are three doctors, and we did not give cards for ones who had to do the control group, and this period of time they could use for the model. Finishing the model, we continue with the scheduled [appointments]” **(E08, Physician, Clinic 3)** . **“** This affects my time, for example, if we finish too late, I have to come back here [to the health center] to include them all in their respective files. So it is more complicated because I have to come back here [to the health center] and sometimes I finish too late to include everything in the system and then include it in all the labs because there [at the location of the sessions] was just the exam and the card. I used to write the signs and other things, but I have to come back here [to the health center] for the medical record, so I use to finish later. The time it took me is a problem. **”(E04, Physician, Clinic 2)** . **“** Almost always we organized our schedules and when we set the date for the control prenatal group we didn't put anything else so that they wouldn't mix or we wouldn't have setbacks, then that day we were completely dedicated to it **” (E10, Nurse, Clinic 4)** .
Patient volume	**“** I'm worried about reducing the number of consultations because that's all I do that day [referring to the GPC sessions] and my minimum number of consultations is sixteen **” (E01, Physician, Clinic 1)** . **“** This control is quicker for me because, for example, in a session I see all the twelve pregnant women, so I am not making appointments for them every month and they have to come to get a consultation very early **”(E04, Physician, Clinic 2)** .
Community	Not identified.
**Testimonials attitudinal dimension**	
Motivation	**“** It would be nice, if there was a stimulus to the health personnel (...) some kind of reward, it could be money, it could be books, it could be some situation that we feel will benefit us, so that it will also make us feel more motivated **”(E08, Physician, Clinic 3)** . **“** A group ends in approximately seven months so in seven months you can update and take everything that happened in that group and according to that train or see what you would improve or remove **”(E09, Nurse, Clinic 3)** .
Leadership	**“** The physician cannot participate in the entire workshop (...) because he either gives the consultation or participates in the workshop, the time is not enough to do both, even if the number of people is reduced **”(E01, Physician, Clinic 1)** . **“** Check who was missing and send them a WhatsApp, because I have the WhatsApp group (...) so I knew why somebody was not in the meeting. **”(E02, Nurse, Clinic 1)** .
Acceptability	**”** It is more comfortable [referring to giving the consultation at the health center] than there [referring to the place where the GPC sessions are held] I don't see the objective that instead of raising the level of attention (...) lowering it. For me, to have to go there to give the consultation [referring to the place where the GPC sessions are held] it is lowering the level **”(E01, Physician, Clinic 1)** . **“** (…) getting closer to the patients, I think the patients perceive it, gives them more confidence and they feels more comfortable, because when you go to the doctor you feel a little worried and here you are closer and welcome **”(E08, Physician, Clinic 3)** .
Address problems	**“** The time there [referring to the place where the GPC sessions are held] is not enough for me, because what I manage to do is measure the fetal cardiac frequency, and pass on the records, explain them too quickly, but when they had finished the part of the workshop, I still had four or five patients left **” (E01, Physician, Clinic 1)** . **“** The day that it is the doctor's turn to do the group control (...) no cards were given, and that period of time is the one that we occupy for, for the model, finishing the model. Because we follow it with the programmed ones that start from eleven o'clock then that is the way that we find **”(E08, Physician, Clinic 3)** .
Work atmosphere	**“** The doctor asked me a lot (...) I told him, “Doctor, I'm going to the group prenatal control” [referring to the doctor's answer] Yes, you're going to have some fun and then they leave me alone here **”(E02, Nurse, Clinic 1)** .
Communication	**“** We have a calendar of activities so the administration knows officially the days we have scheduled and those days we don't have consultation, we are involved since early to organize the area, prepare the things that we will use, we develop the session and, when we finish it, I return to the office to make the medical records and review them **” (E03, Physician, Clinic 2)** . **“** I suppose our director is not convinced and the jurisdiction of the project too, maybe because they don't know it well or because they don't conduct it and obviously it is the first obstacle that limits us **”(E03, Physician, Clinic 2)** . **“** You have to have their authorization for everything, everything that is done here is under the authorization, first of the director of the health center and then of the jurisdiction. So having their authorization and having the material there is no problem **”(E06, Nurse, Clinic 2)** .

## STRUCTURAL

### Space

One of the main challenges was the need for a physical space, allowing around 15 people for the group sessions.

Physical space availability is fundamental, and not all the health centers had space with the ideal characteristics to carry out the group sessions. The space was a critical element during the implementation of the model in Mexico. Specific adaptations were required according to each place (search for spaces near the HC, anticipated reservation of a classroom inside the HC for the sessions, etc.) (TE01 and TE02). However, this required additional staff efforts to move necessary materials and supplies, waiting time if the room was being occupied for other activities; and the need to prepare the room before the beginning of the sessions.

We did not identify any characteristics that would facilitate the implementation of the model in this dimension.

### Resources

The support received by the research team, providing materials and inputs necessary for the group sessions' implementation, favored the development of the model.

The providers highlighted the importance of having the necessary resources for the adequate implementation of the model. However, they said that some health centers did not have enough material and equipment. For example, the units had only one fetal monitoring team (Doppler), which was insufficient to carry out the GPC model activities and, at the same time, the usual activities of the staff who stayed in the health center (TE04).

During our experience implementing the model, the research team provided some of the material (TE11). Since it was not always possible to have resources and equipment assigned exclusively to this type of consultation in the health units, providing the material needed to implement the model is something to consider in future efforts to implement it.

### Health personnel

Uncertainty in the facilitator staff's employment contracts threatened their permanence in the units and the continuity in the implementation of the GPC model. On occasion, implementing the model generated work overload for health personnel and the need to reorganize activities during the working day to comply with the recording of medical and nursing notes and update medical records.

At least two trained team members in each clinic were able to take on the facilitator's role per session at all places. In some cases, the staff's employment contract with the institution was temporary, creating uncertainty about their stay in the units. Ensuring the continuity of the employment's contracts should be considered in future efforts to avoid, as much as possible, the uncertainty surrounding this limitation.

Health care providers expressed readiness to conduct the sessions. Several strategies were implemented to affect the daily dynamics of the health centers as little as possible, such as organizing the GPC in the morning, coordinated teamwork with specific tasks for each member, and scheduling their activities on the consultation day exclusively for the GPC session, without attending to other tasks in the unit (TE08 and TE10).

However, the GPC sessions were generating excess workload for the facilitator staff. They had to reorganize activities during the workday to facilitate the filling out of medical and nursing records. Also, sometimes it was not possible to spend the day exclusively on the GPC session and they had other health unit activities scheduled before or after the sessions (TE04).

### Patient volume

GPC is perceived as a model that favors networking, group cohesion and peer learning. Still, some facilitators perceive that it can also put at risk the daily individual productivity of health personnel.

Bringing together eight to 12 women of similar gestational age to receive prenatal control in the same space was seen as advantageous (TE04). However, some of the facilitator staff perceived that the number of people they care for when they did the GPC was lower compared to standard individual care, reducing the volume of pregnant women they usually serve. This perception is an element that affects the model's acceptance, because of the concern about low individual productivity (TE01).

### Community

For the community category, no testimonies were identified that refer to facilitators or barriers to implementing the GPC model.

## ACTITUDINALS

### Motivation

The GPC model is perceived as a model that motivates the updating the knowledge regarding topics shared in the sessions. Likewise, they identify the lack of stimuli or incentives to the health care personnel who implemented the GPC model.

The testimonies will express the favorable opinion that health personnel have about the educational component of the GPC, which motivates them to prepare themselves better before the session (TE09). However, part of the interviewees considered that, in order to be more motivated, it would be desirable to offer incentives to those who participate in the intervention. The health personnel who worked as facilitators performed additional functions to those, they would typically perform. It would be important to receive some incentive for those additional efforts associated with this model's practice (TE08).

### Leadership

Leadership strategies were created; however, entrenched clinical practice, where health personnel spend a great deal of time on the clinical examination of pregnant women, created some barriers for facilitators to adhere to the model's demands.

The GPC model's implementation requires leadership strategies around the organization, team coordination, monitoring and follow-up of the GPC. Some facilitators reported difficulties in coordinating all session activities during the GPC (TE01).

Operational strategies were implemented for frequent contact with participating pregnant women for follow-up. To this end, health personnel created mechanisms to have greater control over the care of pregnant women. These activities facilitated the leadership of the facilitators. For example, communication mechanisms were created, such as phone calls, messages by WhatsApp, etc. Facilitators offered session scheduling reminders or explored reasons for not attending. They simplified interaction and the creation of an atmosphere of trust between facilitators and pregnant women (TE02).

Difficulties were also identified for health personnel in adjusting to the times during the clinical review. These difficulties generated uncertainty and leadership conflicts during the development of the GPC model or adoption of practices that were far from the essential elements (TE01).

### Acceptability

The GPC model is perceived as a model that favors women's active participation in the care of their pregnancy and prepares them for childbirth and puerperium.

Health personnel found the model acceptable because it facilitated women empowerment and the acquisition of new knowledge to deal with their pregnancy, childbirth, and postpartum periods. Women acquire self-care skills during pregnancy, while at the same time, it is easier to perceive the achievement of a closer and more trusting relationship with health personnel (TE08).

An exceptional testimony considered that some elements of the model decrease the level of quality of care, making explicit the tendency towards the traditional individual consultation style (TE01).

### Address problems

The implementation of the GPC model, at times, generated discomfort because of the time required for the sessions. However, providers implemented alternatives to make up for the time spent implementing the model.

Almost in general, the time dedicated to the sessions was not enough for them to carry out all the activities proposed by the attention model (TE01). However, some solution initiatives were identified, as dedicating the day exclusively to the GPC session without attending to other tasks in the unit to complete the medical notes in the files immediately after the sessions (TE08).

### Work atmosphere

The activities of the model, at times, generated discomfort in the health personnel who were not linked to its implementation in the participating units, who expressed negative comments about the activities and the time allocated to them by the model's facilitators.

At times, there were negative attitudes among some colleagues at the health center towards those implementing the model (TE02). These types of attitudes can generate a feeling of rejection among health center staff not involved in the GPC model. The support of health center managers to facilitate GPC activities is an enabler element of the model. No elements of the work atmosphere were identified to facilitate the implementation of the model.

### Communication

Having a document that justifies leaving the health center to carry out GPC model's activities when it is carried out in another place is useful for the providers. However, it also requires the sensitization of health center directors and senior management.

Having an official communication of the activities to be carried out was useful so that personnel not involved in implementing the model are aware (TE03 *y* TE06).

Having the authorization to implement the model was not enough. Immediate and superior managers must be convinced of the model's benefits to facilitate its implementation (TE03).

Table 3 presents a summary of the main barriers and facilitators found within the health personnel's perceptions who implemented the GPC model.

**Table 3:** Barriers and facilitators perceived by health personnel in the Group Prenatal model in Mexico, 2016-2018.

Structural	Barriers	Facilitators
Category		
Space	Not having suitable spaces to carry out the GPC model sessions.	Not identified.
Resources	The units have only one fetal monitor, which is insufficient to carry out the usual activities of those who stay in the health units and those of the GPC model.	The units were equipped with material provided by the research team to carry out the GPC sessions. (Baumanometer, scale, chaise long, mat, tape measure, sheets and equipment for the different dynamics).
Health personnel	Uncertainty in hiring facilitators. Work overload for both the staff who perform the GPC sessions and for those who stay in the normal consultation.	New strategies were created to perform GPC sessions (organize sessions in the morning, work together, not giving cards in sessions' day).
Patient volume	Low individual productivity on the day of the GPC sessions	Group attention promotes group cohesion and peer learning.
Community	Not identified.	Not identified.
Attitudinal		
Motivation	Lack of incentives to motivate change in the way prenatal care is provided.	The educational component of the GPC motivates them to prepare themselves better, prior to the session, by strengthening the capacity of health personnel to offer health education to pregnant women.
Leadership	Difficulty for the health personnel to adjust to the times of the model, particularly during the clinical review, generating uncertainty and leadership conflicts during the development of the sessions.	It allowed to generate mechanisms to facilitate continuous and timely communication between health personnel and pregnant women (Example: phone calls, WhatsApp messages).
Acceptability	Frequent perception about the comfort of continuing with the traditional model.	The model facilitates the empowerment of pregnant women. Pregnant women acquire greater knowledge about issues of interest and self-care during pregnancy, childbirth and puerperium. Provides closer treatment between provider and patient. It facilitates continuity of care (follow-up by the same health personnel).
Anticipating changes and address problems	Perception that the time allocated to clinical examination during sessions is very little and insufficient to identify signs of alarm and the filling of institutional records.	To avoid work-overload the day of the sessions, the clinics decided that the medical doctors would dedicate exclusively to implementing the model.
Work atmosphere	Negative attitudes of co-workers towards the facilitators.	Support from the managers of the health units for the implementation of the GPC.
Communication	Little knowledge of middle management and health personnel not involved with GPC about the implementation of the GPC model.	To have a document of the Department of Health indicating the activities of the personnel involved in the implementation of the GPC model.

## DISCUSSION

The physical space required to develop the group consultation sessions is one of the main challenges in the Mexican experience. Similar results were identified by Novick et al. ^12^ and Abrams et al. ^10^ , who document the great effort that the programming of spaces in the units represents or that these can be located very far from the workplace or be significantly reduced and uncomfortable spaces for the realization of the sessions ^10^^,^^12^ . This space challenge highlights the limited infrastructure available to health units. Creative strategies are required to implement the GPC, such as using meeting rooms and other spaces near the health centers. The use of the unit's internal meeting room facilitated its implementation. However, the use of spaces out of the medical unit required institutional arrangements, such as transporting medical records out of the unit or moving health personnel, which on many occasions limited the start of the session to the scheduled time.

The implementation of the GPC requires basic inputs and equipment that should be available to the facilitators. It is worth noting the importance of the availability of educational materials according to the characteristics of the women attending the consultation. The provision of supplies and equipment may be a common need in the context of health units in low and middle-income countries, where shortages of these resources are frequently experienced ^14^^,^^26^ . A previous diagnosis is necessary for the eligible units so that the availability of these resources is guaranteed.

The perception of work overload is another barrier that the model imposes on health workers since they also have to respond to patient demand for regular consultation ^14^ . This work overload was centered on the need to reorganize activities during the workday to register medical and nursing records in the Mexican experience. According to national regulations (NOM004 of the medical record), health personnel must make the records and notes corresponding to their intervention when the patient's medical care is offered ^27^ . This barrier has been identified in other studies. Providers report problems in maintaining complete records timely, as the model does not allow records to be filled out during sessions, so they must consider time after the session to do it ^11^ .

GPC is perceived as a model that favors networking, group cohesion and peer learning. However, in the present study, we identified testimonials that expressed the concern of health personnel related to the fact that with GPC, their daily productivity is put at risk. Supplier productivity is not affected ^28^ or may even experience an increase ^9^ . The facilitators in our study explicitly demanded incentives for their participation in the implementation of innovative health care models. There is evidence of experiences giving bonuses to providers, serving as an incentive to increase acceptance of interventions ^29^ . It is important to consider incentives for personnel who participate in interventions because, without compensation, participants may lose their enthusiasm for continuing to participate. The type of incentive must be identified, as not all may be feasible in all cases ^30^ .

The model's implementation favored continuous communication between facilitators and pregnant participants in each group through strategies such as cell phone messages to remember the sessions, ask questions, and report the reason for not attending a session. These activities were a clear expression of the leadership of the facilitators. Our results are similar to those found by Nair et al. ^31^ , showing that communication is essential to increase effectiveness and efficiency in providing health services ^31^ . One of the activities where it was most challenging to maintain the safety and confidence of the facilitators and their leadership was concerning the time spent on clinical review. Similar findings have been reported in other studies that point to barriers such as insecurity about one's ability to facilitate groups or fear of not being a good leader. These conceptions were classified as a “non-intention to change” or initial resistance by health care providers ^11^^,^^17^ . Some of these barriers can be modified in future implementation efforts through ongoing training of health personnel to increase their confidence in the model ^12^ .

The facilitators' good acceptability of the model was evidenced, mainly by recognizing that the model improves their knowledge about patients and the self-efficacy and empowerment of pregnant women. Previous studies have reported similar results; the model allows them to know their patients better ^16^^,^^19^^-^^21^ , to feel that women are more prepared for labor and delivery ^13^^,^^21^ and to appreciate that fathers are better prepared to receive the child ^10^ . They also perceived that women were more inclined to make use of prenatal care ^11^^,^^15^ and that they appreciated not having to wait for their appointment in addition to looking happy ^11^ . Other studies have reported that women became much more involved in the development and care of their pregnancy ^11^^,^^13^^,^^19^^,^^20^ , that they built social networks with each other ^11^^,^^13^^,^^19^^-^^21^ and that they gained self-confidence ^13^^,^^16^ .

Some limitations should be considered regarding our study. We conducted the interviews at different times during the implementation of the model and only one interview per informant. These two elements suggest that the participants' experience concerning implementing the GPC model was not homogeneous at the time of the interview. However, we did not identify differential elements associated with this temporality in the testimonies of the participants.

On the other hand, personnel who agreed to participate in the interviews may have a more favorable attitude toward the GPC than those who did not agree to participate. Additionally, we had more representation from physicians. Despite this, we consider relevant that during the adaptation and implementation of the model in Mexico, we were able to document the experiences of this type of health personnel, which allowed us to identify particular challenges in their participation, of extraordinary importance for future efforts of expansion within the health system.

The participating health centers were intended to care for people without social security, limiting their scope and not explain the phenomenon studied for other population groups and environments. The small number of participating units did not allow us to examine differences related to institutional factors at the site level. Future and more comprehensive studies should explore these factors.

Other researchers found similar limitations to those found in our study. Novick et al. ^12^ noted that personnel who agreed to participate in their study may be more in favor of the GPC model than those who did not agree to participate. Similar research also found limitations that were not identified in our study, such as more respondents during immediate implementation than at late implementation places ^12^ .

The most relevant barriers for the implementation of GPC are focused on the low availability of adequate physical space in the units, the overload of work and the difficulty of adopting a more horizontal relationship with the pregnant women during the group sessions. Concerning the facilitators, they are mainly related to the acceptability that health personnel have of the model for promoting greater participation of pregnant women and greater knowledge of aspects of their self-care. We recommend that future efforts to implement the model focus timely on identified barriers.
