# Supplementary material for: Group Prenatal Care in Mexico: perspectives and experiences of health personnel
Source: Rev Saude Publica. 2020 Nov 27;54:140. doi: 10.11606/s1518-8787.2020054002175 (PMC7703532; doi:10.11606/s1518-8787.2020054002175)
Supplement: Translation

# Translation: Atención Prenatal en Grupo en México: perspectivas y experiencias del personal de salud

**Authors:** Midiam Ibañez-Cuevas, Ileana Beatriz Heredia-Pi, Evelyn Fuentes-Rivera, Zafiro Andrade-Romo, Jacqueline Alcalde-Rabanal, Lourdes Bravo-Bolaños Cacho, Xochitl Guzmán-Delgado, Laurie Jurkiewicz, Blair G Darney

**Affiliations:** I CuernavacaMOR México Instituto Nacional de Salud Pública. Centro de Investigación en Sistemas de Salud. Cuernavaca, MOR, México; II San FranciscoCA USA University of California San Francisco. San Francisco General Hospital. Department of ObGyn & Reproductive Sciences. San Francisco, CA, USA; III PortlandOR USA Oregon Health & Science University. Department of Obstetrics & Gynecology. Portland, OR, USA

**Keywords:** Atención Prenatal., Servicios de Salud Materno-Infantil, organización & administración, Atención Primaria de Salud, Investigación Cualitativa

## Abstract

**OBJETIVO:**

Identificar barreras y facilitadores de la implementación del modelo de Atención Prenatal en Grupo en México (APG), desde la perspectiva del personal de salud.

**MÉTODOS:**

Estudio cualitativo descriptivo en cuatro clínicas de la Secretaría de Salud en dos estados de México (Morelos e Hidalgo) de junio de 2016 a agosto de 2018. Se realizaron 11 entrevistas semi-estructuradas a prestadores de servicios de salud. Se exploraron sus percepciones y experiencias durante la implementación del modelo de APG. Se identificaron barreras y facilitadores para su adopción en dos dimensiones: a) estructurales (espacio, recursos, personal de salud, volumen de pacientes, comunidad) y b) actitudinales (motivación, liderazgo, aceptabilidad, abordaje de problemas, clima y comunicación).

**RESULTADOS:**

Las barreras más relevantes reportadas en el nivel estructural fueron la disponibilidad de espacio físico en las unidades y la sobrecarga de trabajo del personal de salud. Se identificó la dificultad para adoptar una relación menos jerárquica durante la atención a las gestantes en el nivel actitudinal. El principal facilitador a nivel actitudinal fue la aceptabilidad que los prestadores tienen del modelo. Un hallazgo específico para el contexto de la implementación en México fue la resistencia al cambio en la relación médico-paciente; resulta difícil abandonar el modelo jerárquico prevaleciente y cambiar a una relación más horizontal con las gestantes.

**CONCLUSIONES:**

El análisis de la implementación del modelo de APG en México, desde la perspectiva del personal de salud, ha evidenciado barreras y facilitadores similares a las experiencias en otros contextos. Esfuerzos futuros para la adopción del modelo deberán enfocarse en la atención oportuna de las barreras identificadas, sobre todo aquellas señaladas en la dimensión actitudinal que pueden ser modificadas a través de capacitaciones continuas al personal de salud.

## INTRODUCCIÓN

Persisten grandes retos en salud materna en términos de acceso y calidad de la atención en México y en muchos países de la región de América Latina. Las poblaciones principalmente afectadas cuentan con mayor vulnerabilidad estructural y social, con menores coberturas de servicios de salud y menores probabilidades de recibir atención prenatal de calidad ^1^^-^^3^ . El modelo de atención prenatal individual estándar ha sido ampliamente cuestionado debido a los largos tiempos de espera, a las brechas en la continuidad de la atención y a la baja satisfacción de las usuarias con el trato del personal de salud ^4^^,^^5^ .

La Organización Mundial de la Salud ha emitido recomendaciones sobre la atención prenatal para una experiencia positiva durante el embarazo. Las recomendaciones hacen referencia a intervenciones en los sistemas de salud que mejoran la utilización y la calidad de la atención prenatal ^6^ . Se ha sugerido la implementación de un modelo innovador que puede incidir en mejores resultados materno-infantiles en contextos específicos. Se trata del modelo de Atención Prenatal en Grupo (APG), el cual puede ofrecerse como alternativa a la atención prenatal individual. El modelo se caracteriza por integrar componentes de autocuidado, revisión clínica, educación sanitaria y construcción de redes sociales de apoyo ^7^ .

La atención prenatal es otorgada por diversos prestadores de servicios de salud que asumen un rol de facilitador en este modelo. Se organizan grupos de entre ocho y 12 mujeres, con edad gestacional similar (12–20 semanas), conformados en pequeñas cohortes que se reúnen de ocho a 10 veces durante el embarazo, en sesiones de alrededor de dos horas de duración, incorporando todos los componentes mencionados en un circuito de cuatro fases ^8^ . Diversos modelos de APG, incluyendo *CenteringPregnancy* , el modelo grupal más difundido y estudiado, han sido implementados a nivel mundial, tanto en países industrializados como en países de medianos y bajos ingresos, y en poblaciones específicas ^4^^,^^9^^-^^11^ .

Las diferentes fases del ciclo de atención en cada una de las sesiones grupales de la atención prenatal en grupo, tomando como base el modelo *CenteringPregnancy* incluyen: (1) registro y autocuidado: las participantes inician sesión y verifican sus propios signos vitales; (2) socialización o construcción de redes sociales: se sientan en círculo y pasan tiempo conversando libremente con sus compañeras; (3) chequeo médico: en paralelo con la segunda fase, cada mujer tiene una consulta individual con el médico o la partera, que realiza exámenes físicos individuales, en el mismo espacio que el grupo; y (4) educación para la salud: una vez completada la ronda de controles, las mujeres y los facilitadores se integran en un mismo círculo y se les comparte información sobre el embarazo por un enfoque participativo y no jerárquico ( Figura 1 ).

**Figura 1:** Ciclo de atención en el modelo de Atención Prenatal en Grupo. México, 2016–2018.

Fuente: Elaboración propia tomando como referencia al modelo *CenteringPregnancy*^7^^,^^8^ .

El proceso de implementación de intervenciones novedosas como APG enfrenta a barreras importantes que impactan en las etapas de adopción y escalamiento, así como en los resultados que se logran ^12^ . La APG representa un cambio en la relación usuaria-proveedor, así como en la dinámica y organización de las unidades de salud. De ahí la relevancia de explorar las percepciones del personal de salud y su experiencia con la implementación de intervenciones que cambian la forma de ofertar servicios de salud. Esto es fundamental para identificar las barreras y facilitadores de la adopción definitiva del modelo dentro del sistema de salud, y posibles áreas de oportunidad para el fortalecimiento de su puesta en práctica y futuros escalamientos.

Estudios han explorado las barreras y facilitadores de la implementación de la APG, con base en las percepciones del personal de salud que asumió el rol de facilitador. En general, los facilitadores tienen percepciones positivas y consideran que el modelo posee ventajas frente al modelo de atención individual, como mayor tiempo de interacción del personal médico con la gestante, desarrollo de mejores relaciones y mejor comunicación con las mujeres, siendo mucho más gratificante y efectivo para mejorar la promoción de la salud ^13^^-^^19^ .

Entre los retos para el éxito de la implementación del modelo están el apoyo y liderazgo desde el sistema de salud ^18^ . Además, la motivación y comunicación del personal dentro del centro de salud ^12^ y la flexibilidad y compromiso son elementos cruciales para esta etapa ^20^ . Adicionalmente, desde la perspectiva del personal de salud, se han identificado diferentes barreras para la implementación, incluyendo la desconfianza del proceso de revisión clínica en un periodo de alrededor de tres minutos, que de acuerdo a su experiencia es insuficiente ^16^ ; también, falta de espacios físicos adecuados para llevar a cabo las sesiones ^12^^,^^21^ y retos para la calendarización de las fechas, que requiere coordinar los calendarios de las mujeres, del personal de salud y del uso del espacio físico ^11^^,^^18^^,^^20^ .

En México, desde 2016 iniciamos un estudio para adaptar el modelo *CenteringPregnancy* al contexto mexicano y medir la factibilidad y aceptabilidad de las mujeres y del personal de salud durante su implementación inicial ^22^^,^^23^ . Dado que es la primera vez que se implementa el modelo de APG en México, es relevante conocer las experiencias del personal que lo implementó, e identificar qué tanta aceptación tuvo el modelo entre los prestadores de servicios de salud, cuánto se involucraron durante su implementación y el nivel de integración de la nueva intervención al contexto de las unidades de salud.

El objetivo del presente estudio fue identificar barreras y facilitadores de la implementación del modelo de APG en México, desde la perspectiva del personal de salud.

## MÉTODOS

Estudio cualitativo descriptivo realizado en cuatro centros de salud de la Secretaría de Salud, en los estados de Morelos e Hidalgo, México. Exploramos las percepciones del personal de salud para identificar las barreras y facilitadores de la implementación del modelo de APG por medio de entrevistas semiestructuradas. El marco teórico que sirvió de guía para esta investigación fue el propuesto por Novick et al. ^12^ que plantea dos dimensiones de exploración: a) estructurales (espacio, recursos, personal de salud, volumen de pacientes, comunidad.) y b) actitudinales (motivación, liderazgo, aceptabilidad, abordaje de problemas, clima y comunicación). En la Figura 2 , se describen las dimensiones exploradas y las categorías de análisis. Esta propuesta conceptual de análisis se sustentó en la exploración de las percepciones del personal de salud y utilizó como guía los requerimientos o demandas que la implementación del modelo exige a las instituciones y al sistema de salud ( Tabla 1 ). Los factores que favorecen la capacidad del sistema para alcanzar estas demandas fueron identificados como facilitadores, mientras que aquellos que obstaculizan su alcance fueron considerados como barreras de su implementación ^12^ .

**Figura 2:** Marco conceptual y analítico. Dimensiones de exploración y categorías de análisis del modelo de Atención Prenatal en Grupo. México, 2016–2018.

Fuente: Elaborado a partir de Novick et al. ^12^

**Tabla 1:** Elementos esenciales y los requerimientos para el desarrollo del modelo de Atención Prenatal en Grupo. México, 2016–2018.

Elemento esencial	Puntos clave	Elementos requeridos
1. El examen médico se produce dentro del espacio grupal	Organizar algunos elementos de tal manera que el área destinada para el examen médico asegure la privacidad: lugar y nivel del área, música, plantas o algún tipo de división sencilla	Espacio suficiente para mantener una zona que permita hacer los exámenes médicos con privacidad Insumos y equipos para los exámenes físicos (doppler, cinta métrica, formas para recetas, pluma, desinfectante de manos, hojas de referencia, estetoscopio) Recursos para los exámenes físicos (colchoneta o catre y biombo –opcional-) *
2. Las mujeres están involucradas en actividades de auto-cuidado	Las mujeres se toman y registran algunos parámetros de salud como su presión arterial o su peso	Espacio especificado para la auto-valoración de las mujeres Instrumentos de auto-cuidado (báscula, baumanómetro electrónico, pilas, hojas de seguimiento, plumas) * Capacitación previa a las mujeres (instrucciones a personal de salud al respecto)
3. Se utiliza un estilo de liderazgo facilitador	Los facilitadores guían, pero no controlan la discusión y refieren las preguntas al grupo. Las mujeres comparten sus experiencias, sentimientos, ideas e información de manera voluntaria Se establecen reglas para el grupo Los facilitadores se visten de manera casual Las participantes firman un acuerdo de confidencialidad	Material educativo (tarjetas, cajas, tijeras, estambres, rotafolio, plumones, etc.) * Formatos (acuerdo de confidencialidad) * Espacio suficiente para tener sillas para todos los asistentes Sillas acomodadas en círculo Guía de la sesión (plan general o currículo) * Capacitación a personal de salud centrada en estilo de facilitación horizontal
4.Cada sesión tiene un plan general, aunque el énfasis puede variar	Se utilizan los materiales para guiar y evaluar la sesión	Guía de la sesión (plan general o currículo) Capacitación a personal de salud que logre concientizar sobre la flexibilidad del modelo haciendo énfasis en los elementos que no son prescindibles.
5. El grupo se lleva a cabo en un círculo	No hay observadores fuera del círculo El espacio donde se lleva a cabo la sesión es privado Las personas se sientan en círculo en un espacio abierto El inicio de las actividades del círculo no se llevan a cabo hasta que todos los facilitadores y las mujeres se encuentren en el círculo	Espacio suficiente para tener sillas para todos los asistentes Sillas acomodadas en círculo
6. Hay estabilidad en los miembros del grupo incluyendo los facilitadores	Se pueden unir nuevas mujeres al grupo siempre y cuando sea un acuerdo entre los miembros del grupo Los facilitadores están presentes durante toda la sesión Hay un plan por si algún facilitador no puede acudir Si existen estudiantes apoyando en el grupo, estos son supervisados y son consistentes a lo largo de todas las sesiones Los niños de las mujeres no están presentes durante las actividades del círculo	Proceso y estrategias de reclutamiento de mujeres a nivel de facilitadores y de centro de salud Seguimiento a asistencia de las mujeres a las sesiones (recordatorios mediante llamadas o mensajes) Adecuada calendarización de las sesiones para que los facilitadores y mujeres puedan estar presentes toda la sesión Definición de reglas de manera clara con el grupo para determinar aspectos como asistencia de visitantes Capacitación de dos equipos de facilitadores por centro para suplir en caso de algún imprevisto Capacitación a personal de salud que cuente con certidumbre laboral a medianos plazos
7. El tamaño del grupo es óptimo para promover el proceso	Los grupos tienen entre 8-12 mujeres Hay un balance apropiado entre mujeres y facilitadores	Procesos y estrategias de reclutamiento de mujeres a nivel de facilitadores y de centro de salud Seguimiento a asistencia de las mujeres a las sesiones (recordatorios mediante llamadas o mensajes) Adecuada calendarización de las sesiones para que los facilitadores y mujeres puedan estar presentes toda la sesión
8. Se proporciona la oportunidad para la socialización con el grupo	Existe un tiempo libre en el cual las mujeres pueden socializar entre ellas	Capacitación que incluya la relevancia de los procesos de construcción de redes entre mujeres Equipos de facilitadores de al menos dos personas para que mientras uno hace los exámenes médicos el otro promueva la socialización de las mujeres
9. Hay una evaluación continua de los resultados	Hay un reporte de fidelidad, resultados en salud y sostenibilidad del grupo Los facilitadores están preguntando constantemente a las mujeres cómo se sienten con la atención prenatal en grupo	La capacitación deberá incluir elementos sobre continuidad y evaluación constante de progresos y retos

* Material otorgado por el proyecto de investigación.

Los centros de salud (CS) participantes fueron seleccionados según el volumen de pacientes, disponibilidad del espacio físico para implementar el modelo y disponibilidad del personal de salud. Los informantes desempeñaron el rol de facilitadores durante la implementación del modelo. Las entrevistas duraron en promedio 50 minutos y se realizaron cuando el facilitador hubiera concluido la implementación de, al menos, un grupo de APG. Cuatro de los 15 prestadores contactados no aceptaron participar en la entrevista, argumentando no disponer del tiempo suficiente para la realización de ésta.

Utilizamos una guía de entrevista semiestructurada que exploró: a) datos sociodemográficos, b) percepción sobre el modelo, c) barreras y facilitadores para el desarrollo del modelo, d) experiencia como facilitador, y e) sugerencias para mejorar el modelo. La guía fue piloteada con sujetos cercanos a la población objetivo con la intención de lograr la validez interna del instrumento.

Realizamos 11 entrevistas a facilitadores en los centros de salud seleccionados. La mayoría fue realizada a mujeres (8/11). Cinco entrevistas correspondían a personal del área médica, cinco a personal de enfermería y una a personal de trabajo social. Cinco tenían entre 20-29 años de experiencia trabajando en los servicios de salud, cuatro de 10-19 años, y dos entre uno y nueve años.

Las entrevistas fueron transcritas y vertidas en una matriz de respuestas. Clasificamos la información en los componentes temáticos definidos previamente, en correspondencia con el marco conceptual propuesto, para examinar y describir su contenido, según las dimensiones definidas en el marco conceptual.

Realizamos un análisis temático identificando las recurrencias y coincidencias en cuanto a discursos, opiniones y comportamientos reportados. Posteriormente, realizamos el proceso de interpretación de los datos. La comprobación de la consistencia y validez de la información la realizamos por medio de la identificación de los testimonios ofrecidos de manera reiterativa por los participantes. Realizamos un ejercicio de triangulación interpretativa ^24^ de los datos entre dos de las autoras para garantizar su validez ^25^ .

Los participantes firmaron un consentimiento informado escrito autorizando las grabaciones de las entrevistas. El estudio contó con las aprobaciones de los Comités de Ética e Investigación del Instituto Nacional de Salud Pública de México (No. 1756).

## RESULTADOS

Los testimonios (T) que sustentaron los hallazgos identifican al actor citado por medio del número de entrevista ( Tabla 2 ). Iniciamos con un breve resumen del hallazgo central para cada categoría en la presentación de resultados.

**Tabla 2:** Testimonios del personal de salud por dimensión del modelo de Atención Prenatal en Grupo. México, 2016–2018.

Categoría	Testimonios dimensión estructural
Espacio	**“** Si nos vamos a la estructura pues son los espacios (…) de hecho nosotros no contamos con lugar, nos lo prestan, entonces yo creo que eso va a ser una dificultad muy importante (…) un espacio para poder trabajar ambas cosas y pues no sé qué piensen los demás compañeros **” (E01, Médica, Clínica 1)** . **“** Es muy difícil porque no tenemos la infraestructura para, simplemente, poder hacer el taller (…) no tenemos espacio y no nada más este centro de salud, sino muchos centros de salud. Lo principal es la infraestructura” **(E02, Enfermera, Clínica 1)** .
Recursos	**“** Lo del doppler que yo siempre le decía (…) porque nada más tenemos uno entonces luego nos los pedían. No era del control prenatal de grupo era de aquí [del centro de salud] entonces cuando yo me lo llevaba (…) luego iban y lo pedían **”** **(E04, Médica, Clínica 2)** . **“** Aquí tenemos (…) todo el material, las herramientas. Creo que por parte no sé si sea de ustedes o de quien nos mandaron la herramienta que es una caja con todas las cosas [para la realización de las sesiones] **”** **(E11, Enfermera, Clínica 4)** .
Personal de salud	**“** Lo que hicimos que era lo más viable, ocupar el tiempo de la mañana que es cuando se dan las fichas o sea que podemos darlas o no darlas normalmente damos veintiuna fichas somos tres médicos y para cada quien el día que le toca hacer el control de grupo entonces no se daban fichas y ese lapso de tiempo es el que ocupamos para el modelo. Terminando el modelo pues le seguimos con las [citas] programadas **”** **(E08, Médico, Clínica 3)** . **“** Se me complica el tiempo, por ejemplo, si terminamos muy tarde me tengo que regresar acá [al centro de salud] a ingresarlas a todas a su expediente entonces así se me hacía más complicado porque tenía que regresar acá [al centro de salud] y a veces terminaba muy tarde para ingresarlas al sistema y luego ingresar todos los laboratorios porque allá [en el lugar de las sesiones] nada más era la exploración y lo de la tarjeta. Anotaba los signos y eso, pero ya para la nota medica tenía que regresar acá [al centro de salud] entonces si terminaba más tarde. El tiempo eso si se me complicaba **”** **(E04, Médica, Clínica 2)** . **“** Casi siempre nosotros, organizamos nuestros tiempos y cuando poníamos la fecha para el control prenatal en grupo no poníamos nada más para que no se cruzaran o no tuviéramos contratiempos, entonces ese día lo agarrábamos completo **”** **(E10, Enfermera, Clínica 4)** .
Volumen de pacientes	**“** A mí me preocupa bajar el número de las consultas porque ese día nada más me dedico a eso [refiriéndose a las sesiones de APG] y mi mínimo de consultas son dieciséis **”** **(E01, Médica, Clínica 1)** . **“** Este control es más rápido para mí porque, por ejemplo, en una sesión pues yo veo a todas las doce embarazadas ya no las estoy citando cada mes y que tengan que venir a sacar consulta desde muy temprano **”** **(E04, Médica, Clínica 2)** .
Comunidad	No se identificaron.
**Testimonios dimensión actitudinal**	
Motivación	**“** Valdría la pena, si hubiera un estímulo hacia el personal de salud (…) algún tipo de recompensa, puede ser dinero, pueden ser libros, puede ser alguna situación que sintamos en beneficio, para que le dé a uno también más ganas **”** **(E08, Médico, Clínica 3)** . **“** Un grupo termina aproximadamente en siete meses entonces en siete meses te puedes actualizar y llevar todo lo que paso en ese grupo y de acuerdo a eso capacitarte o ver qué mejorarías o que quitarías **”** **(E09, Enfermera, Clínica 3)** .
Liderazgo	**“** El médico no puede participar en todo el taller (…) porque o da la consulta o participa en el taller o sea el tiempo no alcanza como para hacer las dos cosas aun cuando se reduzca el número de personas **”** **(E01, Médica, Clínica 1)** . **“** Revisar quién faltó y enviarles un WhatsApp, porque tengo el grupo de WhatsApp (…) entonces yo sabía por qué no había ido **”** **(** **E02, Enfer** **mera, Clínica 1)** .
Aceptabilidad	**“** Es más cómodo [refiriéndose a dar la consulta en el centro de salud] que allá [haciendo referencia al lugar donde se realizan las sesiones de APG] yo no veo el objetivo de que en lugar de subir de nivel de atención (…) bajarlo, para mí es bajar el nivel tener que irme allá a dar la consulta [haciendo referencia al lugar donde se realizan las sesiones de APG] **”** **(E01, Médica, Clínica 1)** . **“** (…) acercarse al paciente, creo que lo percibe el paciente, le da más confianza y se siente más a gusto, porque uno cuando va al médico como que va así medio receloso y aquí es como más cercano y más cálido **”** **(E08, Médico, Clínica 3)** .
Abordaje de problemas	**“** El tiempo allá [refiriéndose al lugar donde se realizan las sesiones de APG] no me alcanza pues lo que logro hacer es medir la frecuencia cardiaca fetal, y pasar los registros, explicarles demasiado rápido pero cuando ellos ya terminaban la parte del taller a mí todavía me faltan cuatro, cinco pacientes **”** **(E01, Médica, Clínica 1)** . **“** El día que le toca al médico hacer el control de grupo (…) no se daban fichas y ese lapso de tiempo pues es el que ocupamos para, para el modelo terminando el modelo pues le seguimos con las programadas que empiezan a partir de las once entonces esa es la manera que encontramos **”** **(E08, Médico, Clínica 3)** .
Clima	**“** El doctor me reclamaba mucho (…) le decía doctor ya me voy a ir al control prenatal de grupo [hace referencia a la respuesta del médico] si, tú te vas de pachanga mientras luego a mí me dejan solo **”** **(E02, Enfermera, Clínica 1)** .
Comunicación	**“** Tenemos un calendario de actividades entonces ya la dirección conoce por oficio lo que son los días que tenemos programados y esos días ya no nos pasan consulta, nos dedicamos desde temprano a lo que es organizar el área, preparar lo que son las cosas que vamos a utilizar en el tema, desarrollamos la sesión y terminando, regreso a mi consultorio para hacer las notas médicas y revisión de expedientes **”** **(E03, Médico, Clínica 2)** . **“** No está convencida nuestra directora y supongo que también la jurisdicción del proyecto, tal vez porque no lo conocen bien o porque no lo llevan ellos y obviamente es el primer obstáculo como que nos limita **”** **(E03, Médico, Clínica 2)** . **“** Hay que tener la autorización de ellos para todo, todo lo que se hace aquí es bajo la autorización, primero de la directora del centro de salud y después de la jurisdicción. Entonces tendiendo la autorización de ellos y habiendo el material no hay ningún problema **”** **(E06, Enfermero, Clínica 2)** .

## ESTRUCTURALES

### Espacio

Uno de los principales retos fue la necesidad de un espacio físico que permita la presencia de alrededor de 15 personas para las sesiones grupales.

La disponibilidad de espacio físico es fundamental, y no todos los centros de salud contaban con un lugar con las características ideales para realizar las sesiones grupales. Esto fue un elemento crítico durante la implementación del modelo en México. Se requirieron adecuaciones especificas según cada sitio (búsqueda de espacios cercanos al CS, reservación anticipada de un aula dentro del CS para las sesiones, etc.) (TE01 y TE02). Sin embargo, esto demandó esfuerzos adicionales del personal para el traslado de materiales e insumos necesarios, tiempo de espera en caso de que el local estuviera ocupado para otras actividades, y necesidad de acondicionarlo antes de iniciar las sesiones.

No identificamos alguna característica que facilitara la implementación del modelo en esta dimensión.

### Recursos

El apoyo recibido por parte del equipo de investigación, facilitando materiales e insumos necesarios para la implementación de las sesiones grupales, favoreció el desarrollo del modelo.

Los prestadores resaltaron la importancia de contar con los recursos necesarios para la adecuada implementación del modelo. Sin embargo, manifestaron que algunos centros de salud no contaban con suficiente material y equipo. Por ejemplo, en las unidades sólo había un equipo para el monitoreo fetal ( *doopler* ), el cual resultaba insuficiente para realizar las actividades del modelo de APG y, al mismo tiempo, las actividades habituales del personal que se quedaba en el centro de salud (TE04).

Durante nuestra experiencia en la implementación del modelo, parte del material fue proporcionado por el equipo de investigación (TE11). Eso es algo a considerar en futuros esfuerzos para implementar este modelo, dado que no siempre fue posible disponer de insumos y equipos asignados exclusivamente a esta consulta, en las unidades de salud.

### Personal de salud

La incertidumbre en las contrataciones laborales del personal facilitador amenazó su permanencia en las unidades y la continuidad en la implementación de la APG. En ocasiones, la implementación del modelo generó sobrecarga de trabajo en el personal de salud, y la necesidad de reorganizar las actividades durante la jornada laboral para poder cumplir con el registro de las notas médicas y de enfermería, así como actualizar los expedientes clínicos.

Se logró que al menos dos miembros del equipo capacitado en cada clínica adoptasen el rol de facilitadores por sesión en todos los sitios. En algunos casos, el contrato laboral del personal con la institución era de forma eventual, lo que generó incertidumbre sobre su permanencia en las unidades. Esto debe considerarse en futuros esfuerzos para evitar, en lo posible, la incertidumbre que rodea a esta limitante.

Los prestadores de servicios de salud expresaron buena disposición para la realización de las sesiones. Se realizaron varias estrategias para afectar lo menos posible la dinámica diaria de los centros de salud, como organizar la APG en horario matutino, trabajo coordinado en equipo, con tareas específicas para cada integrante, y la programación de sus actividades el día de consulta de manera exclusiva a la sesión de APG, sin atender otras tareas dentro de la unidad (TE08 y TE10).

Sin embargo, las sesiones de APG generaban sobrecarga de trabajo al personal facilitador. Tenían que reorganizar las actividades durante la jornada laboral para facilitar el llenado de notas médicas y de enfermería. Igualmente, en ocasiones se imposibilitaba la opción de destinar el día de manera exclusiva a la sesión de APG programando otras actividades de las unidades de salud antes o después de las sesiones (TE04).

### Volumen de pacientes

La APG se percibe como un modelo que favorece la formación de redes, cohesión de grupo y aprendizaje entre pares, pero algunos facilitadores perciben que también puede poner en riesgo la productividad diaria individual del personal de salud.

Reunir en un mismo espacio ocho a 12 mujeres con edad gestacional similar para recibir el control prenatal fue percibido como algo ventajoso (TE04). Sin embargo, parte del personal facilitador percibió que el número de personas atendidas cuando hacían la APG era menor, en comparación con la atención individual estándar, reduciéndose el volumen de gestantes que ellos atienden habitualmente. Eso es un elemento que afecta la aceptación del modelo, por la preocupación de una baja productividad individual (TE01).

### Comunidad

Para la categoría comunidad no se identificaron testimonios que hagan referencia a facilitadores o barreras de la implementación del modelo de APG.

## ACTITUDINALES

### Motivación

El modelo de APG se percibe como un modelo que los motiva para actualizarse en los temas que se imparten en las sesiones. Así mismo, identifican la falta de estímulos o incentivos al personal participante por los esfuerzos realizados en la implementación del modelo de APG.

Los testimonios expresaran la opinión favorable que tiene el personal de salud sobre el componente educativo de la APG, que los motiva a prepararse mejor previamente a la sesión (TE09). Sin embargo, parte de los entrevistados consideraba que, para estar más motivados, sería deseable ofrecer incentivos a quienes participan en la intervención. El personal de salud que fungió como facilitador realizaba funciones adicionales a las que ordinariamente realizaría, y sería importante recibir algún incentivo por esos esfuerzos adicionales asociados a la práctica de este modelo(TE08).

### Liderazgo

Se crearon estrategias de liderazgo; sin embargo, la práctica clínica arraigada, en la que el personal de salud emplea amplio margen de tiempo para la exploración clínica de las embarazadas, generó algunas barreras para que los facilitadores se apegaran a las exigencias del modelo.

La implementación del modelo de APG requiere de estrategias de liderazgo en torno a la organización, coordinación del equipo, monitoreo y seguimiento de la APG. Algunos facilitadores refirieron dificultades para coordinar todas las actividades de las sesiones durante la APG (TE01).

Se implementaron estrategias operativas para el contacto frecuente con las gestantes participantes para el seguimiento. Para ello, el personal de salud creó mecanismos para tener mayor control sobre la asistencia de las gestantes. Estas actividades facilitaron el liderazgo de los facilitadores. Por ejemplo, se crearon mecanismos de comunicación como llamadas telefónicas, mensajes por WhatsApp, etc. Los facilitadores ofrecían recordatorios de programación de las sesiones o exploraban las razones de no asistencia. Eso facilitó la interacción y la creación de un ambiente de confianza entre facilitadores y gestantes (TE02).

Así mismo, se identificó dificultad para que el personal de salud se ajustara a los tiempos durante la revisión clínica. Eso generó, en ocasiones, incertidumbre y conflictos de liderazgo durante el desarrollo de éstas o adopción de prácticas ajenas a los elementos esenciales (TE01).

### Aceptabilidad

El modelo de APG es percibido como un modelo que favorece la participación activa de las mujeres en el cuidado de su embarazo, y las prepara para el parto y puerperio.

El personal de salud consideraba aceptable al modelo porque facilitaba el empoderamiento y la adquisición de nuevos conocimientos para enfrentar su embarazo, parto y puerperio. Las mujeres adquieren habilidades para realizar su autocuidado durante el embarazo, a la vez que facilita que se perciba el logro de un trato más cercano y de confianza con el personal de salud (TE08).

Un testimonio de excepción consideraba que algunos elementos del modelo bajan el nivel de calidad de la atención, haciendo explicita su preferencia hacia el estilo de consulta tradicional individual (TE01).

### Abordaje de problemas

La implementación del modelo de APG, en ocasiones, generó molestias por el tiempo que hay que dedicar a las sesiones. Sin embargo, los prestadores implementaron alternativas para subsanar el tiempo que dedicaban a la implementación del modelo.

De forma casi generalizada, el tiempo dedicado a las sesiones no les era suficiente para realizar todas las actividades que propone el modelo de atención (TE01). Sin embargo, se identificaron algunas iniciativas de solución, por ejemplo, destinar el día exclusivamente a la sesión de APG sin atender otras tareas dentro de la unidad, para que pudieran completar las anotaciones médicas en los expedientes, inmediatamente posterior al término de las sesiones (TE08).

### Clima

Las actividades del modelo, en ocasiones, generaron molestias en el personal de salud que no estaba vinculado con su implementación en las unidades participantes, quienes se manifestaban con comentarios negativos sobre las actividades y el tiempo destinado a ellas, por parte del personal facilitador del modelo.

En ocasiones, había actitudes negativas por parte de algunas compañeras o compañeros en el centro de salud hacia quien participaba en la implementación del modelo (TE02).

Este tipo de actitudes pode generar un clima de rechazo entre el personal del centro de salud no involucrado en la APG. El apoyo de los directivos de los centros de salud para facilitar las actividades de APG es un elemento facilitador del modelo. No se identificaron elementos del clima laboral que faciliten la implementación del modelo.

### Comunicación

Contar con un documento que justifique la salida del centro de salud para realizar las actividades del modelo de APG, cuando se lleva a cabo en otro sitio, es de utilidad para los prestadores. Sin embargo, también se requiere la sensibilización de directivos de los centros de salud y mandos superiores.

Contar con una comunicación oficial de las actividades a desempeñar fue de utilidad para que el personal que no participa en la implementación del modelo esté enterado (TE03 *y* TE06).

Contar con la autorización para la implementación del modelo no era suficiente. Jefes inmediatos y superiores deben estar convencidos de los beneficios del modelo, para que se facilite su implementación (TE03).

La Tabla 3 presenta un resumen de las principales barreras y facilitadores encontradas dentro de las percepciones del personal de salud que implementó el modelo de APG.

**Tabla 3:** Barreras y facilitadores percibidos por el personal de salud del modelo de Atención Prenatal en Grupo. México, 2016–2018.

Categoría	Barreras	Facilitadores
Estructurales		
Espacio	No contar con espacios idóneos para realizar las sesiones del modelo de APG	No identificados.
Recursos	Las unidades cuentan con un solo equipo para el monitoreo fetal, siendo insuficiente para realizar las actividades habituales de los que se quedan en las unidades de salud y las del modelo de APG.	Las unidades fueron dotadas de material proporcionado por el equipo de investigación para la realización de las sesiones de APG. (Baumanómetro electrónico, bascula, cheslón, colchoneta, cinta métrica, sabanas y equipo para las diferentes dinámicas)
Personal de salud	Incertidumbre en las contrataciones del personal facilitador. Sobrecarga de trabajo tanto para el personal que realiza las sesiones de APG, como para el que se queda en la consulta normal.	Se crearon nuevas estrategias para poder realizar las sesiones de APG (organizar las sesiones por la mañana, trabajar en equipo, no dar fichas el día de las sesiones).
Volumen de pacientes	Baja productividad individual el día que se realizan las sesiones de APG.	La atención grupal favorece la cohesión de grupo y el aprendizaje entre pares.
Comunidad	No identificadas.	No identificados.
Actitudinales		
Motivación	Falta de incentivos que motiven el cambio en la forma de dar la atención prenatal.	El componente educativo de la APG los motiva a prepararse mejor, previo a la sesión, fortaleciendo la capacidad del personal de salud para ofrecer educación sanitaria a las gestantes.
Liderazgo	Dificultad para que el personal de salud se ajuste a los tiempos del modelo, particularmente durante la revisión clínica, generando incertidumbre y conflictos de liderazgo durante el desarrollo de las sesiones.	Permitió generar mecanismos para facilitar la comunicación continua y oportuna entre el personal de salud y las embarazadas (Ejemplo: llamadas telefónicas, mensajes WhatsApp).
Aceptabilidad	Percepción frecuente sobre la comodidad de continuar con el modelo tradicional.	El modelo facilita el empoderamiento de las gestantes. Las gestantes adquieren mayor conocimiento sobre temas de interés y autocuidado durante el embarazo, parto y puerperio. Proporciona un trato más cercano entre proveedor y paciente Facilita la continuidad de la atención (seguimiento por el mismo personal de salud).
Anticipación a cambios y abordaje de problemas	Percepción de que el tiempo destinado a la exploración clínica durante las sesiones es muy poco e insuficiente para identificar signos de alarma y el llenado de los registros institucionales.	Para que el día que se daban las sesiones no se complicara, decidieron no dar fichas a los médicos ese día y dedicarlo exclusivamente al modelo.
Clima laboral	Actitudes negativas de compañeros de trabajo, hacia los facilitadores.	Apoyo de los directivos de las unidades de salud para la implementación de la APG.
Comunicación	Poco conocimiento de mandos medios y personal de salud no involucrado con APG sobre la implementación del modelo de APG.	Contar con un oficio de la secretaria de salud indicando las actividades del personal que participa en la implementación del modelo de APG.

## DISCUSIÓN

El espacio físico que se requiere para desarrollar las sesiones de consulta grupales es uno de los principales retos en la experiencia mexicana. Similares resultados fueron identificados por Novick et al. ^12^ y Abrams et al. ^10^ que documentan el gran esfuerzo que representa la programación de los espacios en las unidades, o que éstos pueden ubicarse muy alejados del lugar de trabajo, o tratarse de espacios muy reducidos y poco confortables para la realización de las sesiones ^10^^,^^12^ . Eso pone en evidencia la limitada infraestructura de la que disponen las unidades de salud. Se requiere de estrategias creativas para implementar la APG como el uso de salas de reuniones y espacios de otras instancias cercanas al centro de salud. La utilización de la sala de reuniones internas en la unidad facilitó su implementación. Sin embargo, el uso de espacios fuera la unidad requirió de arreglos institucionales como el transporte de historias clínicas fuera de la unidad o desplazamiento del personal de salud, lo cual limitó el inicio de la sesión según el tiempo programado en muchas ocasiones.

La implementación de la APG requiere de insumos y equipos básicos de los que deben disponer los facilitadores. Se destaca la disponibilidad de materiales educativos acorde a las características de las mujeres que asisten a la consulta. La aportación de insumos y equipos puede ser una necesidad común en el contexto de las unidades de salud de países de medianos y bajos ingresos, donde la escasez de estos recursos se experimenta frecuentemente ^14^^,^^26^ . Es necesario un diagnóstico previo en las unidades elegibles, de forma que se garantice la disponibilidad de estos recursos.

La percepción de sobrecarga de trabajo es otra de las barreras que el modelo impone a los trabajadores de salud, ya que además tienen que responder a la demanda de los pacientes de la consulta regular ^14^ . Esta sobrecarga de trabajo estuvo centrada en la necesidad de reorganizar las actividades durante la jornada laboral para poder registrar las notas médicas y de enfermería, en la experiencia mexicana. Según la normatividad nacional (NOM004 del expediente clínico), el personal de salud deberá hacer los registros y/o anotaciones correspondientes a su intervención durante el tiempo en que se ofrece la atención médica del paciente ^27^ . Esta barrera se ha identificado en otros estudios. Los proveedores reportan tener problemas para mantener los expedientes completos, en los tiempos adecuados, ya que el modelo no permite hacer el llenado durante las sesiones, por lo que deben considerar tiempo posterior a la sesión para realizarlo ^11^ .

La APG se percibe como un modelo que favorece la formación de redes, cohesión de grupo y aprendizaje entre pares. Sin embargo, en el presente estudio identificamos testimonios que expresaban la preocupación del personal de salud relacionada con el hecho de que con la APG se pone en riesgo su productividad diaria individual. La productividad de los proveedores no se ve afectada ^28^ o incluso puede experimentar un incremento ^9^ . Así mismo, los facilitadores en nuestro estudio demandaron explícitamente incentivos por su participación en la implementación de modelos de atención a la salud innovadores. Hay evidencia sobre experiencias con la entrega de bonos a los prestadores, sirviendo como incentivo para aumentar la aceptación de intervenciones ^29^ . Es importante considerar los incentivos para el personal que participa en las intervenciones ya que, sin recompensas, los participantes pueden perder su entusiasmo en seguir participando. Así mismo, hay que identificar el tipo de incentivo, ya que no todos pueden ser factibles en todos los casos ^30^ .

La implementación del modelo favoreció la comunicación continua entre facilitadores y gestantes participantes en cada grupo, a través de estrategias como mensajes en el celular para recordar sobre las sesiones, reportar el motivo de no acudir a alguna sesión o preguntar dudas. Estas actividades fueron una clara expresión del liderazgo realizado por los facilitadores. Nuestros resultados son similares a los encontrados por Nair et al. ^31^ , evidenciando que la comunicación es importante para incrementar la eficacia y la eficiencia en la provisión de los servicios de salud ^31^ . Una de las actividades donde resultó más difícil mantener la seguridad y confianza de los facilitadores y su liderazgo fue en relación al tiempo destinado a la revisión clínica. Hallazgos similares se han reportado en otros estudios que señalan barreras como inseguridad sobre sus propias habilidades para facilitar grupos y/o miedo a no ser un buen líder. Estas concepciones fueron clasificadas como una “no intención al cambio” o resistencia inicial por parte de los proveedores de servicios de salud ^11^^,^^17^ . Algunas de estas barreras pueden ser modificadas, en futuros esfuerzos de implementación, a través de capacitaciones continuas al personal de salud que incrementen su confianza en el modelo ^12^ .

Se evidenció buena aceptabilidad al modelo por los facilitadores, fundamentalmente al reconocer que el modelo mejora su conocimiento sobre sus pacientes y la autoeficacia y empoderamiento de las gestantes. Resultados similares han sido reportados por estudios previos, en los que se reporta que el modelo les permite conocer mejor a sus pacientes, ^16^^,^^19^^-^^21^ sentir que las mujeres están más preparadas para el trabajo de parto y parto ^13^^,^^21^ y apreciar que los padres están más preparados para la crianza ^10^ . También percibieron que las mujeres estaban más dispuestas a hacer uso de la atención prenatal ^11^^,^^15^ y que apreciaban no tener que esperar por su cita además de que se observaban felices ^11^ . Otros estudios han reportado que las mujeres se involucraron mucho más en el desarrollo y cuidado de su embarazo, ^11^^,^^13^^,^^19^^,^^20^ que construyeron redes sociales entre ellas ^11^^,^^13^^,^^19^^-^^21^ y que ganaron confianza en ellas mismas ^13^^,^^16^ .

Limitaciones deben ser consideradas en el presente estudio. Las entrevistas las realizamos en diferentes momentos de la implementación del modelo y aplicamos solamente una entrevista por informante. Estos dos elementos sugieren que la experiencia que el personal de salud participante tenía al momento de la entrevista, con relación a la implementación del modelo de APG, no era homogénea entre todos los participantes. Sin embargo, no identificamos elementos diferenciales asociados a esta temporalidad en los testimonios de los participantes.

Por otra parte, el personal que accedió a participar en las entrevistas podría tener una actitud más favorable hacia la APG que quienes no aceptaron participar. Adicionalmente, tuvimos mayor representación de médicos. A pesar de ello, consideramos que es relevante que durante la adaptación e implementación del modelo en México pudiéramos documentar las experiencias de este tipo de personal de salud, lo que permitió identificar retos muy puntuales de su participación, de extraordinaria importancia para futuros esfuerzos de escalamiento dentro del sistema de salud.

Los centros de salud participantes estuvieron destinados a la atención de personas sin seguridad social, lo que limita su alcance y no permite explicar el fenómeno estudiado para otros grupos poblacionales y entornos. El pequeño número de unidades participantes no hizo posible que examináramos las diferencias entre los sitios, relacionadas con factores institucionales a nivel de sitio. Futuros estudios de mayor alcance deberán explorar estos factores.

Otras investigaciones encontraron limitaciones parecidas a las encontradas en nuestro estudio. Novick et al. ^12^ señalaron que el personal que aceptó participar en su estudio podría estar más a favor del modelo de APG que los que no aceptaron participar. Así mismo, investigaciones similares encontraron limitaciones que en nuestro estudio no se identificaron, como más entrevistados durante la implementación inmediata, que en los sitios de implementación tardía ^12^ .

Las barreras más relevantes para la implementación de la APG se focalizan en la poca disponibilidad de espacio físico adecuado en las unidades, la sobrecarga de trabajo y la dificultad de adoptar una relación más horizontal con las gestantes durante las sesiones grupales. Con relación a los facilitadores, principalmente se relacionan con la aceptabilidad que el personal de salud tiene del modelo por propiciar una mayor participación de las gestantes y un mayor conocimiento de aspectos de su autocuidado. Se recomienda que futuros esfuerzos de implementación del modelo se enfoquen en la atención oportuna de las barreras identificadas.
